# Description of a new *Barsine* Walker, 1854 from India and Nepal (Lepidoptera, Erebidae, Arctiinae, Lithosiini)

**DOI:** 10.3897/zookeys.941.51344

**Published:** 2020-06-16

**Authors:** Anton V. Volynkin, Navneet Singh, Jagbir Singh Kirti, Harvinder Singh Datta

**Affiliations:** 1 Altai State University, Lenina Avenue, 61, RF-656049, Barnaul, Russia Altai State University Barnaul Russia; 2 National Research Tomsk State University, Lenina Avenue, 36, RF-634050, Tomsk, Russia National Research Tomsk State University Tomsk Russia; 3 Zoological Survey of India, M-Block, New Alipore, Kolkata, 700053, West Bengal, India Zoological Survey of India Kolkata India; 4 Department of Zoology and Environmental Sciences, Punjabi University, Patiala, Punjab, 147002, India Punjabi University Patiala India

**Keywords:** Asia, *Barsine kirata*, *B. valvalis* Kaleka, *B. thomasi* Kaleka, new species, new synonymy, red and yellow forms

## Abstract

A new species, *B. kirata* Volynkin & N. Singh, **sp. nov.**, similar to *B. germana*, is described from India and Nepal. The existence of two colour forms in some species of the genus *Barsine* Walker, 1854 is revealed. A new synonymy is established for *Barsine germana* (Rothschild, 1913), which includes two forms that were described as three different species: *Barsine germana* (Rothschild, 1913) (the yellow form) = *B. valvalis* Kaleka, 2003, **syn. nov.**, and *B. thomasi* Kaleka, 2003, **syn. nov.** (the red-spotted forms).

## Introduction

Until recently, *Barsine* Walker, 1854 was considered to be a very large and polyphyletic genus including more than a hundred valid species (Holloway 2001; Kaleka 2003, 2018; Černý and Pinratana 2009; Bucsek 2012, 2014; Dubatolov et al. 2012; Dubatolov and Bucsek 2013; Wu et al. 2013; Kirti and Singh 2015, 2016; Černý 2016; Volynkin and Černý 2016a, b, c, 2017a, b, c, d, 2018a, b, 2019; Bayarsaikhan et al. 2018; Huang et al. 2018, 2019; Joshi et al. 2018; Spitsyn et al. 2018; Volynkin 2018; Volynkin et al. 2018, 2019a, b, c, d). Volynkin et al. (2019e) separated several lineages into distinct genera, and now *Barsine* includes 65 species and five subspecies of its type species, *B. defecta* Walker, 1854, having a basal saccular process.

**Figures 1–10.**
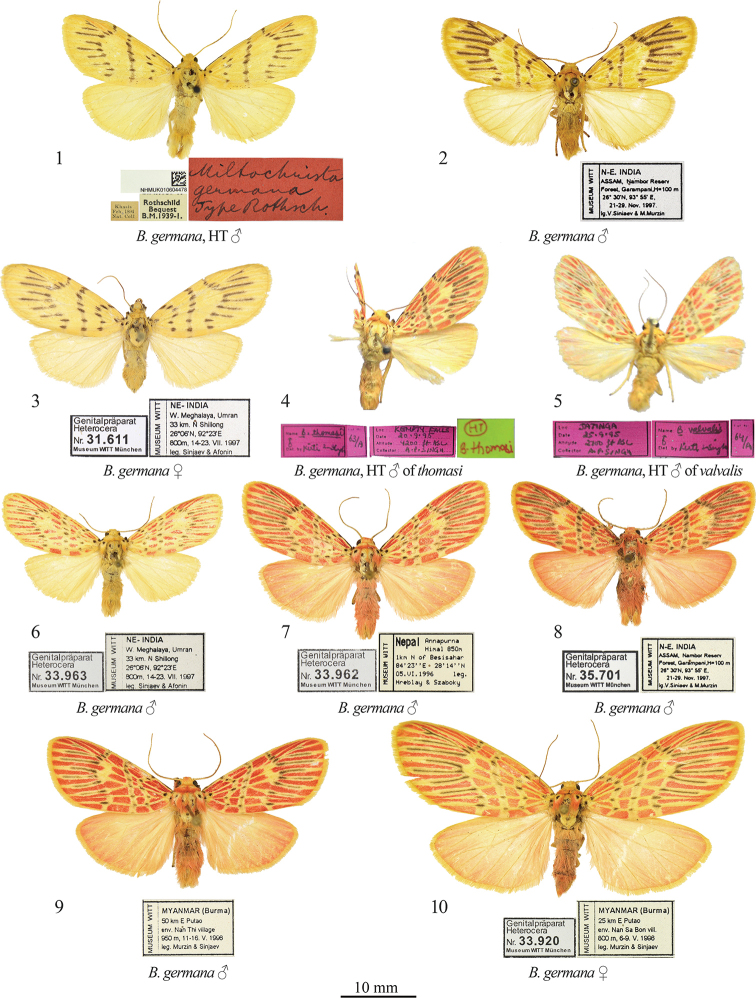
*Barsine germana*: adults **1** holotype male, NE India (NHMUK) **2** male, northeastern India (MWM/ZSM) **3** female, northeastern India (MWM/ZSM) **4** Holotype male of *B. thomasi* (NZCZSI) **5** holotype male of *B. valvalis* (NZCZSI) **6** male, northeastern India (MWM/ZSM) **7** male, C Nepal (MWM/ZSM) **8** male, northeastern India (MWM/ZSM) **9** male, northern Myanmar (MWM/ZSM) **10** female, northern Myanmar (MWM/ZSM).

Dissections of numerous specimens of various species of *Barsine* displayed the existence of two colour forms in some of them: the common form having reddish forewing pattern elements together with black ones, and the yellow form lacking reddish forewing pattern elements. The latter, yellow form is usually very rare and has so far been found only in *B. defecta* (Figs 19, 20), *B. orientalis bigamica* Černý, 2009 (illustrated by Bayarsaikhan et al. 2018), *B. gratissima* (de Joannis, 1930), *B. obsoleta* (Reich, 1937), and *B. cacharensis* N. Singh & Kirti, 2016. In some species, intraspecific variation is high and expressed not only in the presence or absence of red pattern elements but also in the shade of the ground colour and red spots, the size of the red and black pattern elements, body size, and even forewing shape (Figs 1–10). Such polymorphism is obvious evidence of a polygenic inheritance, and this matter needs extensive molecular study.

The red and yellow forms of some species have been described as distinct species, as in the case of *Barsine germana* (Rothschild, 1913) (the yellow form; Figs 1–3), and *B. valvalis* Kaleka, 2003 and *B. thomasi* Kaleka, 2003 (the red-spotted form; Figs 4–10). In the present paper, we synonymize *B. valvalis* and *B. thomasi* with *B. germana*. In addition, dissections of red-spotted specimens of this group from various regions of Nepal and India revealed the existence of two species very similar externally but clearly different in their genitalia structures. One of them is described below.

## Materials and methods

Abbreviations of the depositories used: **NHMUK** = Natural History Museum (formerly British Museum of Natural History, London, UK); **NZCZSI** = National Zoological Collection, Zoological Survey of India (Kolkata, India); **MWM/ZSM** = The Bavarian State Collection of Zoology (Museum Witt München / Zoologische Staatssammlung München, Munich, Germany); **ZFMK** = Zoological Research Museum Alexander Koenig (Zoologisches Forschungsmuseum Alexander Koenig, Bonn, Germany).

The genitalia of specimens deposited in NHMUK, MWM/ZSM, NZCZSI, and ZFMK collections were dissected, stained with eosin B and mounted in Euparal on glass slides using standard methods of preparation (Lafontaine and Mikkola 1987; Fibiger 2007). Photographs of imagos deposited in NHMUK and MWM/ZSM were taken using a Nikon D3100/AF-S camera equipped with a Nikkor 18–55 mm lens. Genital preparations made by A.V. Volynkin were photographed with the same camera attached to a microscope with an LM-scope adapter.

## Taxonomic part

### Barsine germana

Taxon classificationAnimaliaLepidopteraErebidae

(Rothschild, 1913)

DBA09F93-22B8-5AFD-A9C7-5CDF4907C000

Figs 1–10 , 21–24 , 29 , 30

Miltochrista germana Rothschild 1913: 214 (type locality: [India, Meghalaya, the Khasi Hills] “Khasia Hills, Assam”).
Barsine valvalis Kaleka 2003: 97, figs A, 12–19 (type locality: [India] “Assam: North Cachar Hills, Jatinga”), syn. nov.
Barsine thomasi Kaleka 2003: 100, figs B, 25–32 (type locality: [India, Uttarakhand] “Uttar Pradesh: Kempty falls”), syn. nov.

#### Type material examined.

***Holotype*** of *Miltochrista germana* (by monotypy) (Fig. 1): male, red handwritten label “*Miltochrista germana* Type Rothsch.” / printed label “Khasis, Feb. 1894, Nat. Coll.” / printed label “Rothschild Bequest B.M. 1939–1.” / printed label with QR-code “NHMUK010604478” (Coll. NHMUK). ***Holotype*** of *Barsine valvalis* (Figs 5, 24): male, lilac label “Loc. Jatinga | Date 25.9.95 | Altitude 2700 ft. A.S.L. | Collector A.P. Singh” / lilac label “64/ A” / lilac label “Name *B. valvalis* | ♂ | Det. by Kirti & Singh”, gen. prep. by H.S. Datta (Coll. NZCZSI). ***Holotype*** of *Barsine thomasi* (Figs 4, 23): male, lilac label “Loc. Kempty Falls | Date 20.9.95 | Altitude 4200 ft. A.S.L. | Collector A.P. Singh” / lilac label “63/ A” / lilac label “Name *B. thomasi* | ♂ | Det. by Kirti & Singh” / lettuce green label “HT | *B. thomasi*”, gen. prep. by H.S. Datta (Coll. NZCZSI).

#### Other material examined.

**India.** 1 male, Khasis, Oct. 1896, Nat. Coll., slide NHMUK010313291 Volynkin (Coll. NHMUK); 1 female, Khasia Hills. Assam / Rothschild Bequest B.M. 1939–1., slide NHMUK010313292 Volynkin (Coll. NHMUK); 1 female, NE India, W Meghalaya, Garo Hills, Nokrek National Park, 25°40'N, 91°04'E, 1150 m, 2–13.VII 1997, leg. Afonin & Sinyaev (Coll. MWM/ZSM); 28 males, 19 females, NE India, W Meghalaya, Umran, 33 km N Shillong, 26°06'N, 92°23'E, 800 m, 14–23.VII.1997, leg. Sinyaev & Afonin, slides MWM 31610, MWM 33963 (males), MWM 31611 (female) Volynkin (Coll. MWM/ZSM); 21 males, 11 females, NE India, Assam, Nameri Nat. Park, 40 km N Tezpur, 150 m, 27°20'N, 93°15'E, 24.VII–2.VIII.1997, leg. Sinyaev & Murzin, slides MWM 33964 (male), MWM 33965 (female) Volynkin (Coll. MWM/ZSM); 74 males, 12 females, NE India, Assam, Nambor Reserve Forest, Garampani, h = 100 m, 26°30'N, 93°56'E, 21–29.XI.1997, leg. V. Sinyaev & M. Murzin, slides MWM 31612, MWM 31617, MWM 33922, MWM 35701 (males), MWM 31613, MWM 33923, MWM 35703 (females) Volynkin (Coll. MWM/ZSM); 5 males, NE India, Arunachal Pr., Etalin vicinity, 28°36'56"N, 95°53'21"E, 700m, 12–25.V.2012, L. Dembický & O. Šauša leg., slide MWM 35704 Volynkin (Coll. MWM/ZSM); 4 males, [NE India] Assam: Haflong: Jatinga, 01.X.[19]95 (Coll. NZCZSI). **Nepal**: 21 males, 2 females, Nepal, Annapurna Himal, Geirigan village, 1340 m, 28°20'N, 83°45'E, 25.VI.1996, leg. Gy. M. László & G. Ronkay, slides MWM 33949 (male), MWM 33950 (female) Volynkin (Coll. MWM/ZSM); 6 males, 1 female, Nepal, Annapurna Himal, 1000m, 1 km S of Bahundanda, 28°20'N, 84°25'E, 06.VI.1996, leg. Hreblay & Szaboky (Coll. MWM/ZSM); 1 male, Nepal, Annapurna Himal, Ulleri, 1900 m, 28°23'N, 83°43'E, 3.X.1994, leg. Csorba & Ronkay (Coll. MWM/ZSM); 1 male, Nepal, Annapurna Himal, 850 m, 1 km N of Besisahar, 28°14'N, 84°23'E, 05.VI.1996, leg. Hreblay & Szaboky, slide MWM 33962 Volynkin (Coll. MWM/ZSM). **Myanmar**: 45 males, 18 females, Myanmar (Burma), 50 km E Putao, env. Nan Thi village, 950 m, 11–16.V 1998, leg. Murzin & Sinyaev, slide MWM 33921 (male) Volynkin (Coll. MWM/ZSM); 53 males, 17 females, Myanmar (Burma), 25 km E Putao, env. Nan Sa Bon village, 800 m, 6–9.V.1998, leg. Murzin & Sinyaev, slides MWM 33919 (male), MWM 33920 (female) Volynkin (Coll. MWM/ZSM); 21 male, 2 females, Myanmar (Burma), 21 km E Putao Nan Sa Bon village 550 m, 1–5.V.1998, leg. Murzin & Sinyaev (Coll. MWM/ZSM).

#### Remarks.

Joshi et al. (2018) considered this species to consist only of yellow-patterned individuals matching the holotype (Figs 1–3). Nonetheless, dissections of similarly patterned red-spotted syntopic specimens (Figs 4–10) revealed these two color forms to be conspecific. The red-patterned form had been described twice before by Kaleka (2003) as *B. thomasi* Kaleka, 2003 (Figs 4, 23) and as *B. valvalis* Kaleka, 2003 (Figs 5, 24). These names are therefore synonymized here with *B. germana*.

The holotype of *B. germana* is undissected. However, the senior author has microscopically examined the tips of its valvae, which have the distal saccular process structure identical to those in the holotypes of *B. valvalis* and *B. thomasi*. The holotype is also externally similar to specimens from the same region of India, and clearly different from *B. kirata*, sp. nov. A detailed comparison of *B. germana* with *B. kirata* sp. nov. is provided below.

*Barsine germana* varies considerably in its size: the forewing length is 13–17 mm in males and 16–23 mm in females.

#### Distribution.

Northern (Uttarakhand) and northeastern India (Meghalaya, Assam, Arunachal Pradesh) (Rothschild 1913; Kaleka 2003; Joshi et al. 2018), eastern Nepal, and northern Myanmar (Kachin state).

### Barsine kirata

Taxon classificationAnimaliaLepidopteraErebidae

Volynkin & N. Singh sp. nov.

E1664E7B-58AC-5CE7-AF02-059ABB0916D4

http://zoobank.org/487A79AB-5ACC-44D7-BDE8-DBB8187FFB41

Figs 11–18 , 25–28 , 31 , 32

#### Type material.

**Figures 11–20.**
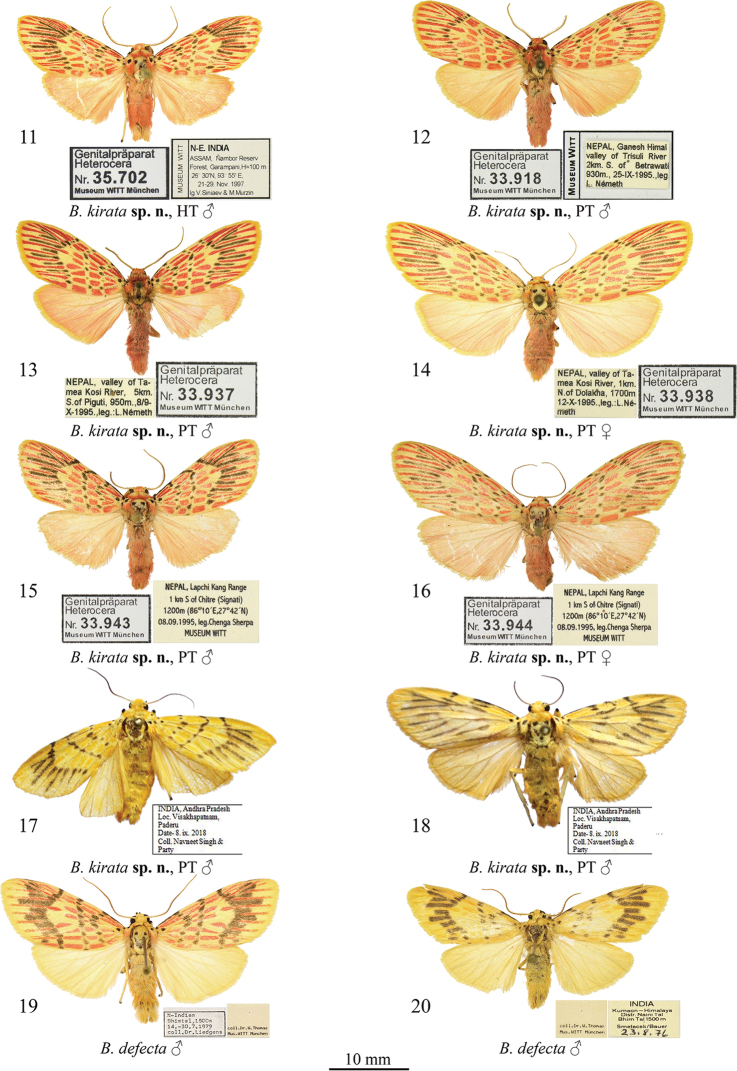
*Barsine* spp.: adults **11–18***B. kirata* sp. nov. **11** holotype male, northeastern India (MWM/ZSM) **12** paratype male, Nepal (MWM/ZSM) **13** paratype male, Nepal (MWM/ZSM) **14** paratype female, Nepal (MWM/ZSM) **15** paratype male, Nepal (MWM/ZSM) **16** paratype female, Nepal (MWM/ZSM) **17** paratype male, southeastern India (NZCZSI) **18** paratype male, southeastern India (NZCZSI) **19, 20***B. defecta*: **19** male, N India (MWM/ZSM) **20** male, N India (MWM/ZSM).

***Holotype*** (Figs 11, 25): male, “N-E. India, Assam, Nambor Reserv[e] Forest, Garampani, H = 100 m, 26°20'N, 93°55'E, 21–20. Nov. [IX] 1997, leg. V. Siniaev & M. Murzin”, slide MWM 35702 Volynkin (Coll. MWM/ZSM).

***Paratypes*. India**: 1 male, same data as in the holotype (Coll. MWM/ZSM); 6 males, India, Andhra Pradesh, Visakhapatnam, Paderu, 08.IX.2018, leg. Navneet Singh & Party, gen. preps by H.S. Datta (Coll. NZCZSI); **Nepal**: 1 male, Nepal, Tanahoun distr., Baisakhe Ghat, 10 km W Duleguunda, 630 m, 10.X.1994, leg. Csorba & Ronkay, slide MWM 33961 Volynkin (Coll. MWM/ZSM); 2 females, Nepal, valley of Tamea Kosi River, 1 km N of Dolakha, 1700 m, 12.X.1995, leg. L. Németh, slide MWM 33938 Volynkin (Coll. MWM/ZSM); 1 male, Nepal, valley of Tamea Kosi River, 5 km S of Piguti, 950m, 8/9.X.1995, leg.: L. Németh, slide MWM 33937 Volynkin (Coll. MWM/ZSM); 2 males, 19 females, Nepal, Lapchi Kang Range, 1 km S of Chitre (Signati), 1200m, (27°42'N, 86°10'E), 08.09.1995, leg. Chenga Sherpa, Museum Witt, slides MWM 33943 (male), MWM 33944 (female) Volynkin (Coll. MWM/ZSM); 3 males, 5 females, Nepal, Tanahoun distr., Bimalnager village, 530 m, 12.X.1994, leg. Csorba & Ronkay (Coll. MWM/ZSM); 7 males, 1 female, Nepal, Ganesh Himal, valley of Trisuli River, 2 km S of Betrawati, 930 m, 25.IX.1995, leg. L. Németh, slide MWM 33918 (male) Volynkin (Coll. MWM/ZSM); 2 males, Nepal, Ganesh Himal, 1040 m, Mailung Khola, ca 20 km NE Trisuli, 28°04'5"N, 85°12'5"E, 24.IX.1995, leg. B. Herczig & Gy. M. László (Coll. MWM/ZSM); 1 male, Nepal, Royal Chitwan National Park, Island Jungle Resort, 240 m, 21–23.VI.1993, leg. M. Hreblay, G. Csorba (Coll. MWM/ZSM).

#### Remarks.

Kirti and Singh (2016) erroneously recorded this species from India as *B. orientalis bigamica* Černý, 2009. Like *B. germana*, *B. kirata* sp. nov. is dimorphic, but the yellow form (Figs 17, 18) is rare, and, so far, known only from the state of Andhra Pradesh (southeast India).

#### Diagnosis.

**Figures 21–24.**
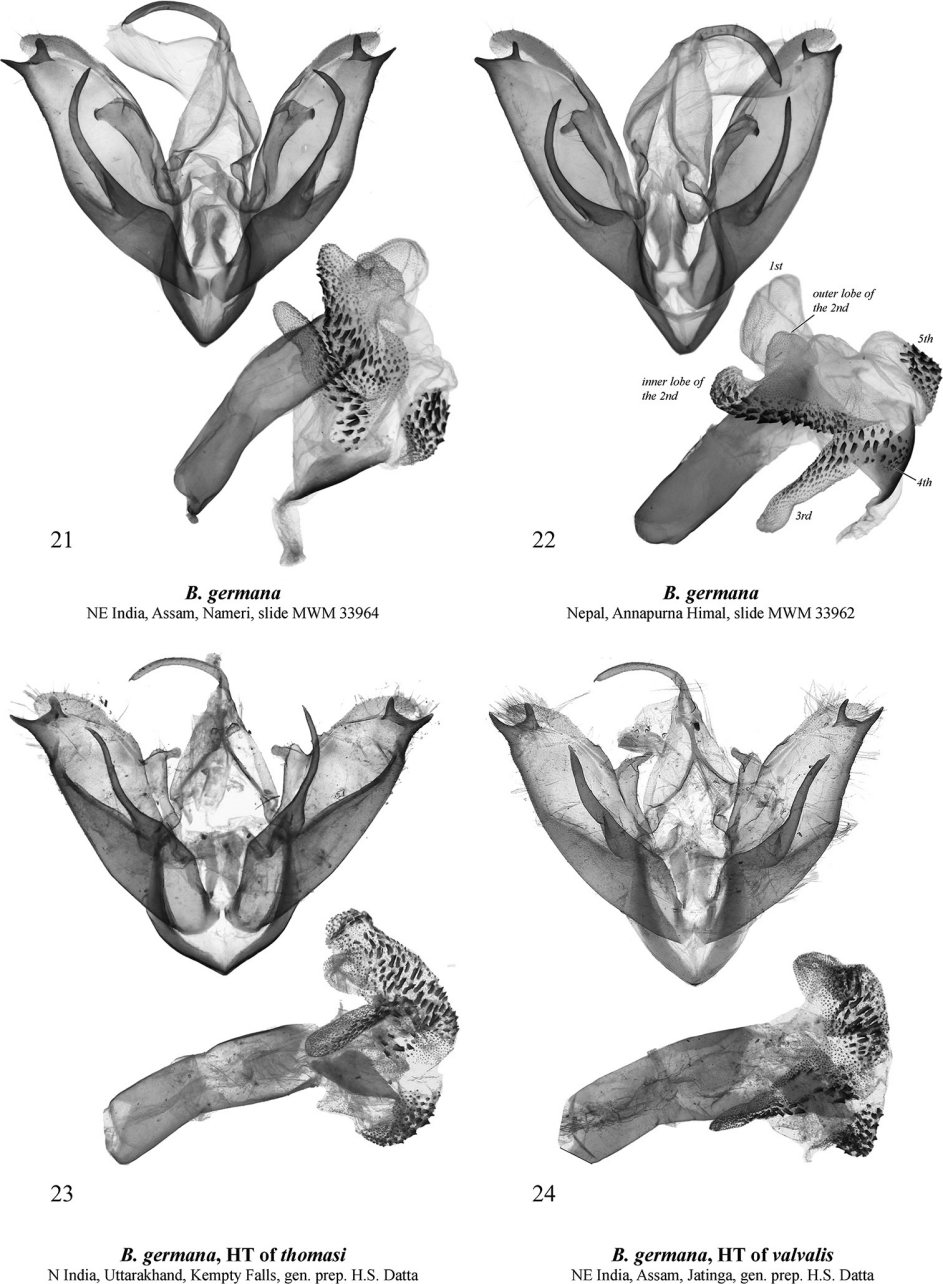
*Barsine germana*: male genitalia **21** northeastern India, slide MWM 33964 Volynkin **22** Nepal, slide MWM 33962 Volynkin **23** holotype of *B. thomasi*, northern India, prep. H.S. Datta **24** holotype of *B. valvalis*, northeastern India, prep. H.S. Datta.

**Figures 25–28.**
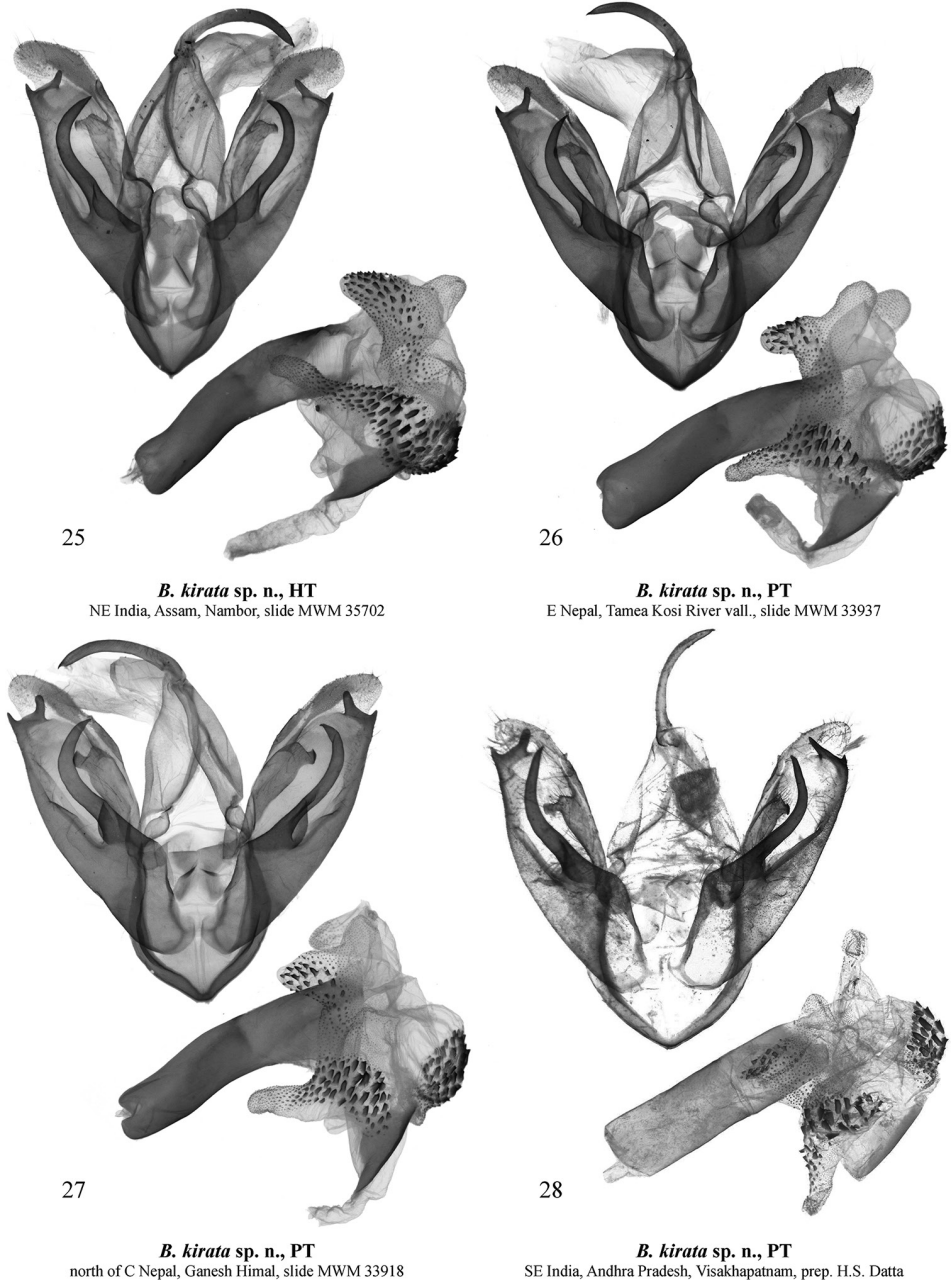
*Barsine kirata* sp. nov., male genitalia **25** holotype, northeastern India, slide MWM 35702 Volynkin **26** paratype, eastern Nepal, slide MWM 33937 Volynkin **27** paratype, central Nepal, slide MWM 33918 Volynkin **28** paratype, southeastern India, prep. H.S. Datta.

The new species (Figs 11–18) is very similar externally to *B. germana* (Figs 1–10) and can be distinguished from it by its less wavy antemedial transverse line. The male genital capsule of the new species (Figs 25–28) differs clearly from that of *B. germana* (Figs 21–24) by the distal ventral process of the valva having a short distal lobe directed dorso-distally and the longer dorsal lobe dorsally directed, while in *B. germana* the distal lobe is more elongated and distally directed and the dorsal lobe is dorso-distally directed. Additionally, in *B. kirata* sp. nov. the juxta is broader than that of *B. germana*, the basal saccular process is stouter and more curved, the distal lobe of valva is larger, and the distal part of the distal ventral process of valva is more robust. The vesica of *B. kirata* sp. nov. differs from that of *B. germana* by its slightly narrower 1^st^ medial diverticulum, the smaller cornuti on the 2^nd^ medial diverticulum, and the slightly less elongated 3^rd^ medial diverticulum. The female genitalia of the new species (Figs 31, 32) clearly differ from those of *B. germana* (29, 30) by the significantly shorter ductus bursae with shorter subostial folds, the wrinkled posterior sclerotised section of corpus bursae, the slightly smaller signum, the presence of the second, band-like signum in the anterior section of corpus bursae (absent in *B. germana*), and the slightly smaller lateral membranous protrusion of the corpus bursae.

#### Description.

***External morphology of adults*** (Figs 11–18). Wingspan 14.5–16 mm in males (15 mm in holotype) and 18–20 mm in females. Male antennae ciliate, female antennae filiform, pale ochreous in both sexes. Head crimson with yellow spot on frons. Thorax yellow, with three black dots; collar and tegulae yellow with crimson margins. Forewing broad with slightly elongated and rounded apex. Forewing ground colour yellow, with a pattern of black dots and strokes and various-shaped crimson spots and strokes between veins; costa between base and antemedial line black; basal spot very small, black; subbasal spot black, round; antemedial line W-like wavy, black, interrupted into a series of variously shaped, small spots on veins; medial line almost straight, angled inwards at costa, interrupted into a series of variously shaped small spots on veins; postmedial line smoothly curved outwards medially, presented as a series of black thin strokes of different lengths between veins; cilia amber yellow. Hindwing pale pink with yellowish suffusion along veins; cilia amber yellow along outer margin and apex, and pink along anal margin. Yellow form of species lacks all reddish pattern elements. Abdomen pink with admixture of yellow scales. ***Male genitalia*** (Figs 25–28). Tegumen moderately broad, shorter than valva; vinculum short but robust, V-shaped with convex lateral margins. Valva massive, with almost parallel margins; medial costal process broadly trigonal, with convex outer margin and slightly broadened and blunted tip; distal costal process very small, tubercle-shaped; distal lobe of valva large, oblique; sacculus broad, its basal process robust, broad, curved dorsally, apically rounded, reaches the distal costal process; distal ventral process broad, bilobate, its dorsal lobe approximatly two 2 times longer than distal lobe, narrow, apically blunted, directed dorsally; distal lobe short, thorn-shaped, directed dorso-distally. Uncus narrow, laterally flattened, curved, medially broadened, with claw-like tip; tuba analis broad. Scaphium narrow, weakly sclerotized. Juxta weakly sclerotized, X-shaped, with broader apical lobes. Aedeagus elongated, narrow, slightly curved medially and broadened distally. Vesica membranous, short and broad, with several diverticula: 1^st^ medial diverticulum elongated, sack-like with rounded tip, its distal half weakly granulated; 2^nd^ medial diverticulum bilobate, its inner lobe covered with numerous variously sized short but robust cornuti, outer lobe weakly granulated; 3^rd^ medial diverticulum long, covered with numerous variously sized short but robust trigonal cornuti; 4^th^ medial diverticulum small, globular, covered with small trigonal cornuti; 5^th^ medial diverticulum broadly globular, its outer surface with broad cluster of small, trigonal cornuti of various sizes; basal diverticulum absent; distal plate of vesica broad, trigonal with slightly convex outer margin, heavily sclerotized. ***Female genitalia*** (Figs 31, 32). Ostium bursae broad. Ductus bursae dorso-ventrally flattened, sclerotized, its lateral margins more weakly sclerotized than medial part; posterior section of ductus bursae slightly broadened, with several narrow longitudinal subostial folds. Corpus bursae broad, sac-like, with posterior section moderately sclerotized with wrinkled posterior margin ventrally, and reniform signum dorsally; border between posterior and anterior sections of corpus bursae weakly sclerotized, with a band of short scobination; anterior section of corpus bursae thick and membranous, with a band-like signum surrounded by a rugose area. Appendix bursae weakly sclerotized and granulated, short, conical, situated postero-laterally, directed posteriorly and curved inwards. Apophyses long and thin, apophyses posteriores thinner and ca 1.8 times longer than apophyses anteriores. Papillae anales broad, trapezoidal, weakly setose.

#### Distribution.

The new species is known from northeastern India (Sikkim, Darjeeling, and Assam) (Kirti and Singh 2016, as *B. orientalis bigamica*), southeastern India, and Nepal (present study).

**Figures 29–32.**
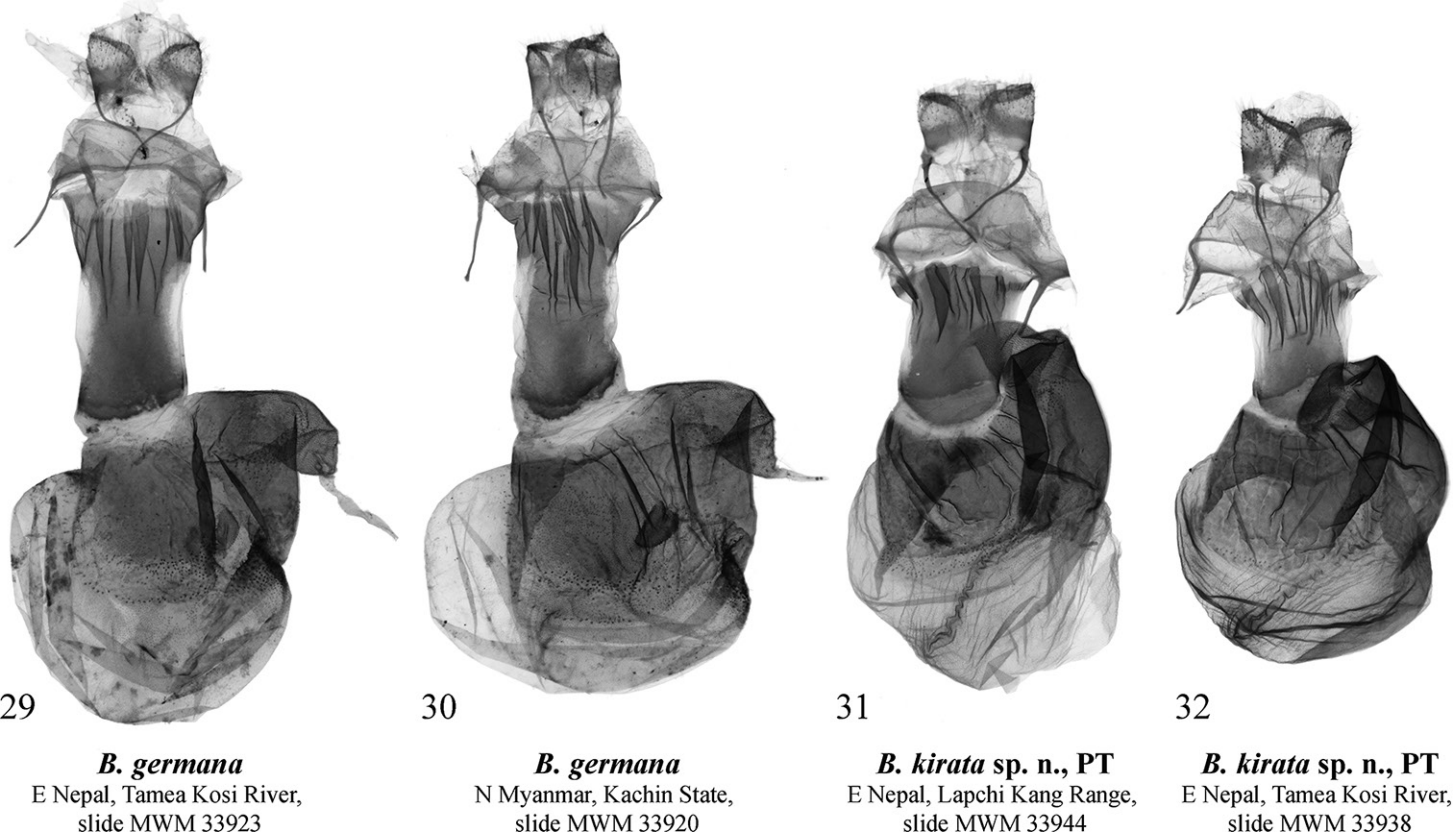
*Barsine* spp.: female genitalia **29***B. germana*, Nepal, slide MWM 33923 Volynkin **30***B. germana*, northern Myanmar, slide MWM 33920 Volynkin **31***B. kirata* sp. nov., paratype, Nepal, slide MWM 33944 Volynkin **32***B. kirata* sp. nov., paratype, Nepal, slide MWM 33938 Volynkin.

#### Etymology.

The Kirata are the people inhabiting the Himalayas and northeastern India.

## Supplementary Material

XML Treatment for Barsine germana

XML Treatment for Barsine kirata
